# Risk factors for low back pain in the Chinese population: a systematic review and meta-analysis

**DOI:** 10.1186/s12889-024-18510-0

**Published:** 2024-04-26

**Authors:** Qiang Li, Leyun Peng, Yiding Wang, Yonghui Yang, Zongbao Wang

**Affiliations:** 1 Shuguang-Anhui Hospital Affiliated to Shanghai University of Traditional Chinese Medicine, Anhui University of Chinese Medicine, No. 45 Shihe Road, Shushan District, 230000 Hefei, Anhui, People’s Republic of China; 2 School of Medicine, Shandong Xiandai University, No. 20288 Jingshi East Road, Licheng District, 250104 Jinan, Shandong People’s Republic of China; 3 The Second Affiliated Hospital of Anhui University of Chinese Medicine, No. 300 Shouchun Road, Luyang District, 230000 Hefei, Anhui, People’s Republic of China

**Keywords:** Low back pain, Chinese population, Risk factors, Meta-analysis

## Abstract

**Background:**

In China, the world’s largest developing country, low back pain (LBP) is a common public health issue affecting workability. This meta-analysis aimed to systematically assess the risk factors of LBP in the Chinese population.

**Methods:**

Four English language and four Chinese databases were searched, and cross-sectional studies on the risk factors for LBP in Chinese populations were identified and collected. The search timeframe covered the period from the establishment of the database to November 2023. Two researchers independently reviewed the literature, extracted the data, and evaluated the risk of bias. Begg’s and Egger’s tests were used to evaluate publication bias.

**Results:**

Fifteen cross-sectional studies involving 86,575 people were included. Seven risk factors for LBP were identified. Six risk factors were statistically significant: Cigarette smoking (odds ratio [OR] = 1.55; 95% confidence interval [CI]: 1.15, 2.08, *P* = 0.004, I^2^ = 72%), body mass index (BMI) ≥ 28 kg/m² (OR = 4.51; 95% CI: 3.36, 6.07, *P* < 0.00001, I^2^ = 8%), female sex (OR = 1.54; 95% CI: 1.25, 1.90, *P* < 0.0001, I^2^ = 63%), vibration exposure at work (OR = 1.65; 95% CI: 1.16, 2.34, *P* = 0.006, I^2^ = 84%), working overtime (OR = 2.57; 95% CI: 1.12, 5.91, *P* = 0.03, I^2^ = 85%), and lack of exercise (OR = 2.48; 95% CI: 1.62, 3.78, *P* < 0.0001, I^2^ = 0%). One risk factor that was not statistically significant was standing for long periods (OR = 1.02; 95% CI: 0.82, 1.26, *P* = 0.88, I^2^ = 73%).

**Conclusions:**

This study found that smoking, a BMI ≥ 28 kg/m², female sex, vibration exposure at work, working overtime, and lack of exercise may be risk factors for LBP in the Chinese population. Because the included studies were cross-sectional and the certainty of the evidence was very low, the results need to be interpreted cautiously. Multicentre, high-quality studies should be conducted in the future. To reduce the prevalence of LBP, the Chinese government and hospitals must develop early screening programs and implement effective preventive and interventional measures.

**Trial registration:**

This study is registered in the PROSPERO database (No. CRD42023447857).

**Supplementary Information:**

The online version contains supplementary material available at 10.1186/s12889-024-18510-0.

## Background

Low back pain (LBP) is a common symptom of many known and unknown pathologies or diseases [1]. Pain is usually located at the lower edge of the ribs, in the lumbosacral region, or accompanied by unilateral and bilateral lower limb nervous system symptoms [2]. LBP is a health problem that can occur in all age groups. According to literature reports, approximately 90% of people worldwide experience LBP at some stage of their lives [3]. LBP ranks sixth among all disease burdens in developed and developing countries [4, 5]. Hartvigsen et al. reported [1] that the global economic burden of LBP is likely to increase further.

In China, LBP is also a major public health problem. According to Chen et al. [6], the annual prevalence of LBP in Chinese adults is 20.88–29.88%. This is closely related to China’s social and economic conditions. China is a large agricultural country with a population of more than 1.4 billion, and many people perform manual labour work. Furthermore, the level of agricultural mechanisation in China is significantly lower than that in developed countries [7], and the degree of industrial intelligence is also relatively low. As a rapidly emerging developing country, many adults in China work long hours to earn more income for their household expenses in response to escalating costs of living [8]. During their leisure time, the participation rate of Chinese people in sports activities was only 13.8%, which was significantly lower than that in Western countries [9]. The vast differences between Chinese and Western cultures and the lack of medical knowledge in the Chinese population may aggravate the occurrence of chronic pain in the Chinese population [10]. One study found that the prevalence of chronic pain in China was 31.54%, and most patients with chronic pain were in the northern and southern coastal areas [10]. Among the patients with chronic pain, 24.06% did not visit the hospital, and 36.788% did not receive any treatment [10]. Compared with developed Western countries, Chinese people have an insufficient understanding of chronic pain; the medical treatment rate of the affected population is low, and the number of patients undergoing standard treatment is also low [10]. Additionally, there are 1.14 billion smokers worldwide and an estimated 306 million adult smokers in China, the country with the largest number of smokers worldwide [11, 12]. China’s economic development, popularisation of medical knowledge, and lifestyle habits show unique characteristics. These factors jointly affect LBP prevalence and risk in the Chinese population.

The risk factors for LBP reported in the literature can be divided into three aspects: (1) lifestyle factors such as obesity, smoking, and lack of exercise; (2) social factors such as working overtime, physical labour, education, and job satisfaction; and (3) psychological factors such as depression and anxiety [1, 13, 14]. If people have an unhealthy lifestyle, choose to eat junk food, smoke, and are unwilling to exercise, there is an increased risk of LBP. In highly competitive metropolitan cities, employees often work overtime and engage in heavy physical labour, resulting in low job satisfaction and the possibility of LBP. However, psychological factors are often overlooked. A cross-sectional study in South Korea found that the severity of depressive symptoms was associated with an increased risk of LBP [15]. A retrospective study conducted in the United States revealed that a healthy emotional state and a positive lifestyle can aid recovery from LBP [16]. In addition, regarding certain factors such as prolonged standing and education level, the research findings are inconsistent. Research conducted by Li et al. [17] and Jia et al. [18] indicate that prolonged standing may be a protective factor against LBP, whereas research conducted by Peng et al. [19] and Yue et al. [20] shows otherwise. Conflicting research is also seen in education. Xu et al. [21], Liu [22], and Jia et al. [18] reported an association between education and LBP, whereas Ye et al. [23] reported the opposite. The inconsistency in these research results may be related to the significant differences in the industries in which the research participants were located.

Although existing studies have provided valuable knowledge about LBP, few studies on the risk factors of LBP in the Chinese population exist. Investigations and research are limited by the sample size and study population. After reviewing Chinese and English language databases, we could not locate any evidence-based studies on the risk factors for LBP in the Chinese population. This finding suggests a systematic and quantitative approach is required to address this issue. As an evidence-based research method, meta-analysis can comprehensively analyse the results of multiple studies, improve the accuracy of result estimation, and identify and quantify potential risk factors. Therefore, the purpose of this study was to systematically review and integrate existing studies on the risk factors of LBP in the Chinese population through a meta-analysis. The aims were to enhance statistical precision, address inconsistencies among studies, and ensure robust conclusions, thereby providing an evidence-based foundation for formulating effective prevention and intervention strategies.

## Methods

This evidence-based medical study is registered in The International Prospective Register of Systematic Reviews (PROSPERO) database (No. CRD42023447857). The study was conducted in accordance with the Preferred Reporting Items for Systematic Reviews and Meta-Analyses (PRISMA) guidelines [24, 25].

### Search strategy

PubMed, Web of Science, Embase, Cochrane Library, CNKI, WanFang Date, VIP, and CBM databases were searched to collect cross-sectional studies on the risk factors for LBP in the Chinese population. From the time the databases were created until 1 November 2023, eight databases were searched using MeSH Terms, index terms, and keywords. Chinese search terms: ‘low back pain’ or ‘lower back pain’, ‘risk factors’ or ‘influencing factors’ or ‘related factors’. The English language search strategy is detailed in Additional File 1.

### Inclusion criteria

- Research individuals: People with LBP (pain arising from the lower edge of the ribs in the lumbosacral and sacroiliac areas, with or without radiating pain in the lower limbs [2]) in China.
- Exposure factors: Risk factors that may lead to the onset of LBP.
- Outcome measure: Clinical diagnosis confirming the presence of LBP.
- Study type: Cross-sectional studies.
- Studies with original clear odds ratios (ORs) and 95% confidence intervals (95% CIs).
- Language: Chinese and English.

### Exclusion criteria

- Republished studies.
- Studies with different research participants and methods.
- Studies on individuals with lumbar stenosis or other comorbidities.
- Dissertations and conference papers.
- Research lacking data or not including analytical factors.

### Data extraction and quality assessment

Two researchers (Qiang Li and Yiding Wang) with extensive retrieval experience read the literature and extracted the information. The contents of the records included the first author, publication year, sample size, risk factors, and OR (95% CI). The third researcher (Leyun Peng) made the final decision whenever problems were encountered regarding data extraction; quality was evaluated. Two reviewers evaluated cross-sectional studies using the Agency for Healthcare Research and Quality methods [26]. With this method [27], 1–3 points mean low quality, 4–7 points mean medium, and 8–11 points mean high quality. In the Newcastle-Ottawa Scale, 0–4 points mean low quality, 5–6 points mean medium, and 7–9 points mean high quality.

The Grading of Recommendations, Assessment, Development, and Evaluation system [28] was employed to assess the evidence quality for the risk factors. This is based on factors such as study design, risk of bias, imprecision, inconsistency, indirectness, publication bias, and effect size [29–34]. The level of evidence was categorised as high, medium, low, or very low.

### Statistical analyses

To guarantee the accuracy of the pooled effect estimates, we conducted a meta-analysis based solely on risk factors evaluated in a minimum of four studies. We used Revman 5.3 software for statistical analysis. OR (95% CI) was used as the effect analysis statistic. I² was used to test heterogeneity using *P*-values. Low, moderate, and high degrees of heterogeneity were indicated by the I^2^ statistical values of 25, 50, and 75%, respectively [35]. When I^2^ was > 50%, sensitivity analyses were performed to exclude each study individually to determine the stability of the findings. Funnel plot analysis was performed when at least 10 studies were included. Begg’s and Egger’s tests were used to evaluate the publication bias.

## Results

### Study selection

**Fig. 1:**
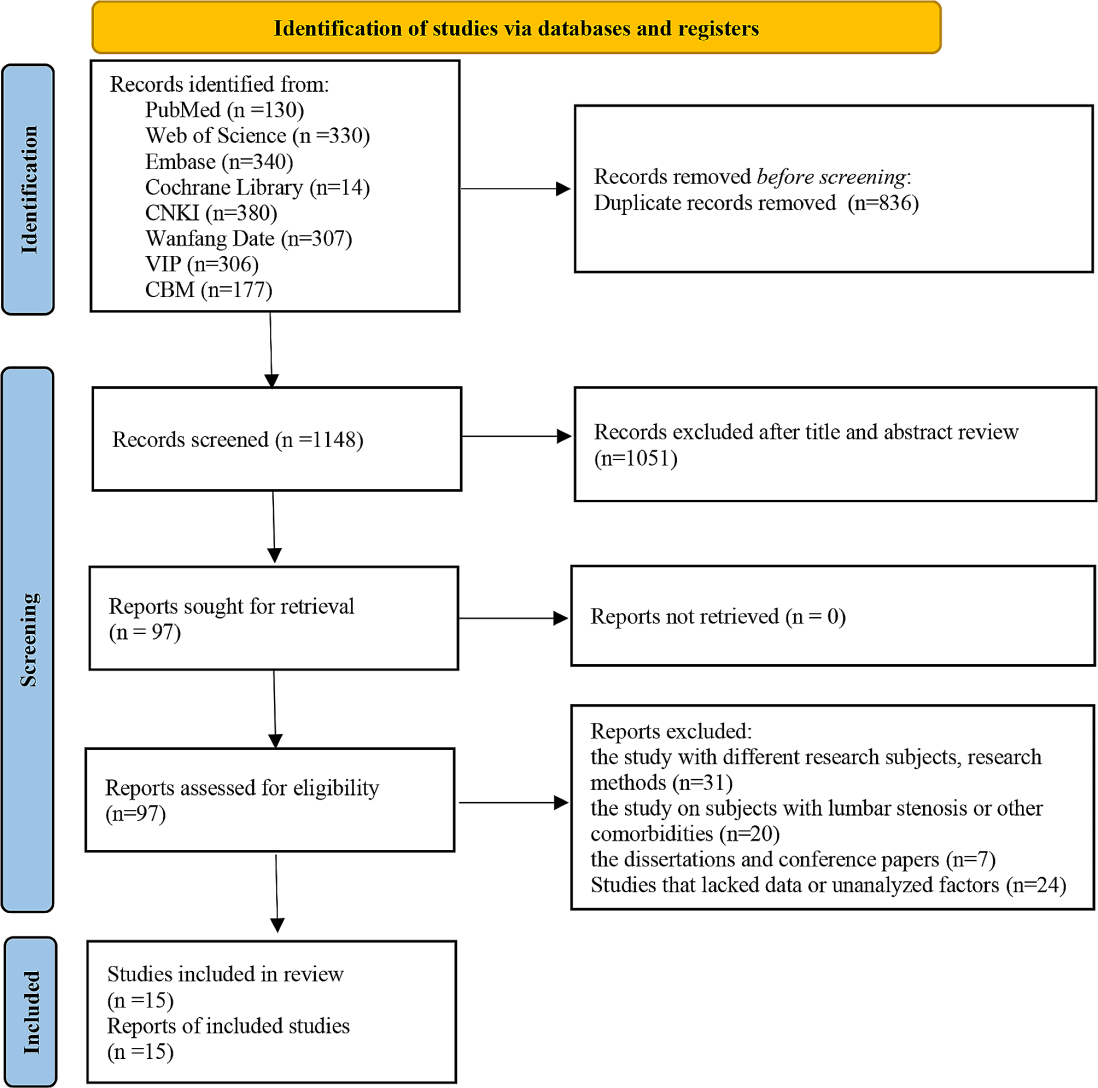
Flow chart of the search process for the studies

We initially searched the database and obtained 1,984 records. In total, 836 duplicate records were deleted using EndNote X9. We then reviewed the titles and abstracts of the literature and excluded 1,051 records. After reading the full texts, 82 studies were excluded. Finally, 15 studies were included for appraisal and analysis. A flow chart of the article screening is shown in Fig. 1. The studies excluded in the final step are presented in Additional File 2.

### Study characteristics and methodologic quality

The basic characteristics and quality assessment results of the included studies are summarised in Table 1. For details of the quality assessment of the literature, please refer to Additional File 3. Fifteen papers [18–20, 22, 36–46] were published between 2006 and 2022. The sample size ranged from 96 to 57,501, with a total of 86,575 individuals. The quality assessment of the 15 cross-sectional studies yielded scores ranging from 8 to 10.

**Table 1:** Baseline characteristics of all included studies

References	Study design	Sample size	Mean age	Diagnostic criteria	Risk factors reported	Quality score
Zhang Yu 2021 [36]	Cross-sectional study	LG: 22; CG: 74	63.21 ± 11.55	Follow the Expert consensus on acute/chronic nonspecific low back pain in China for judgment [47].	Cigarette smoking; BMI ≥ 28 kg/m²; Working overtime	8
Wu Gang 2019 [37]	Cross-sectional study	LG: 142; CG: 323	36.87 ± 4.87	Follow the Expert consensus on acute/chronic nonspecific low back pain in China for judgment [47].	Cigarette smoking; BMI ≥ 28 kg/m²; Lack of exercise	8
Yang Qichang 2020 [38]	Cross-sectional study	LG: 80; CG: 307	NR	Follow the Expert consensus on acute/chronic nonspecific low back pain in China for judgment [47].	Cigarette smoking; BMI ≥ 28 kg/m²; Working overtime; Lack of exercise	8
Wang Xi 2019 [39]	Cross-sectional study	LG: 80; CG: 275	46.45 ± 8.65	Follow the Expert consensus on acute/chronic nonspecific low back pain in China for judgment [47].	BMI ≥ 28 kg/m²; Vibration exposure at work; Lack of exercise	9
Liu Feng 2020 [22]	Cross-sectional study	LG: 39; CG: 292	NR	Follow the Expert consensus on acute/chronic nonspecific low back pain in China for judgment [47].	Female sex; Vibration exposure at work	9
Wang Jiuqing 2022 [40]	Cross-sectional study	LG: 96; CG: 260	NR	Follow the Expert consensus on acute/chronic nonspecific low back pain in China for judgment [47].	Vibration exposure at work; Lack of exercise	8
Peng Banglai 2017 [19]	Cross-sectional study	3800	37.47 ± 6.87	In the past year, there has been pain or limited mobility lasting ≥ 24 h between the rib margin of the back and the folds of the buttocks.	Female sex	8
Jia Ning 2022 [18]	Cross-sectional study	57,501	32.32 ± 9.16	Follow The National Institute of Occupational Safety&Health criteria for musculoskeletal injury for judgment [48].	Cigarette smoking; Vibration exposure at work; Standing for long periods	9
Zhang Qiong a 2019 [41]	Cross-sectional study	LG: 105; CG: 393	31.1 ± 7.6	Follow the Standardised Nordic Musculoskeletal Questionnaire [49], the Dutch Musculoskeletal Questionnaire [50] and the Job Content Questionnaire [51] for judgment.	BMI ≥ 28 kg/m²; Female sex	9
Zhang Qiong b 2019 [41]	Cross-sectional study	519	35.7 ± 6.9	Follow the Standardised Nordic Musculoskeletal Questionnaire [49], the Dutch Musculoskeletal Questionnaire [50] and the Job Content Questionnaire [51] for judgment.	BMI ≥ 28 kg/m²; Female sex	9
Zhang Qiong c 2019 [41]	Cross-sectional study	543	38.4 ± 9.5	Follow the Standardised Nordic Musculoskeletal Questionnaire [49], the Dutch Musculoskeletal Questionnaire [50] and the Job Content Questionnaire [51] for judgment.	BMI ≥ 28 kg/m²	9
Wang M 2017 [42]	Cross-sectional study	719	40.1 ± 5.8	Follow the modified Delphi questionnaire [2] and the Nordic Musculoskeletal Questionnaire [52] for judgment.	Working overtime	9
Yue Pengying 2012 [20]	Cross-sectional study	893	NR	Follow the Standardised Nordic Musculoskeletal Questionnaire [49] and the Dutch Musculoskeletal Questionnaire [50] for judgment.	Female sex; Standing for long periods	9
Barrero Lope H 2006 [43]	Cross-sectional study	13,965	NR	The patient reported experiencing LBP in the past year.	Vibration exposure at work	10
Xu Guangxing 2012 [44]	Cross-sectional study	LG: 997; CG: 540	35.2 ± 16.7	Follow the Standardised Nordic Musculoskeletal Questionnaire [49] and the Dutch Musculoskeletal Questionnaire [50] for judgment.	Working overtime; Standing for long periods	9
Wei Gejin 2018 [45]	Cross-sectional study	2565	20.57 ± 3.44	LBP was defined as pain in the lower back extending from the 12th rib to the lumbar or lumbosacral area that is not related to a tumor, inflammation, injury, or lumbar disc protrusion.	Cigarette smoking	8
Liu Xiaotong 2012 [46]	Cross-sectional study	LG: 786; CG: 1259	NR	In the past three months, there has been LBP that lasts for a whole day or longer.	Cigarette smoking; Female sex	8

LG: LBP group, CG: control group, NR: not reported

### Risk factors for LBP in the Chinese population

Seven risk factors were identified: cigarette smoking, body mass index (BMI) ≥ 28 kg/m², female sex, vibration exposure at work, working overtime, lack of exercise, and standing for long periods.

The certainty of the evidence for these seven risk factors was very low. Some risk factors, such as smoking, vibration exposure at work, and standing for a long time, might have had publication bias. The comprehensive results and the certainty of evidence assessment are presented in Table 2. A detailed rating of the certainty of evidence assessment is provided in Additional File 4. Begg’s and Egger’s tests are presented in Additional File 5.

**Table 2:** Risk factors’ pooled analysis and certainty of evidence assessment

Risk factors	Number of included studies	Pooled effects			Certainty of Evidence Assessment
		OR[95%CI]	*P* value	I^2^	
Cigarette smoking	6	1.55[1.15, 2.08]	0.004	72%	Very Low
BMI ≥ 28 kg/m²	5	4.51[3.36, 6.07]	<0.00001	8%	Very Low
Female sex	5	1.54[1.25, 1.90]	<0.0001	63%	Very Low
Vibration exposure at work	5	1.65[1.16, 2.34]	0.006	84%	Very Low
working overtime	4	2.57[1.12, 5.91]	0.03	85%	Very Low
Lack of exercise	4	2.48[1.62, 3.78]	<0.0001	0%	Very Low
Standing for long periods	4	1.02[0.82, 1.26]	0.88	73%	Very Low

### Cigarette smoking

**Fig. 2:**
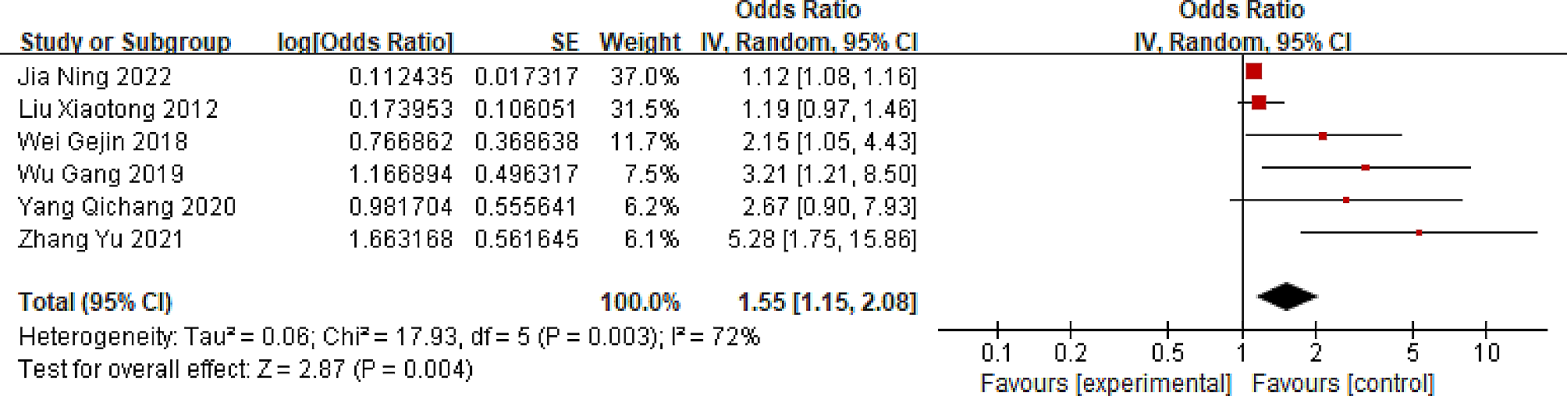
Forest plot of cigarette smoking

Six studies assessing the correlation between smoking and LBP were included in this meta-analysis [18, 36–38, 45, 46]. Comprehensive findings suggest that individuals in China who smoke are at a heightened risk of experiencing LBP compared with those who do not smoke (OR = 1.55; 95% CI: 1.15, 2.08, *P* = 0.004, I^2^ = 72%) (Fig. 2). Sensitivity analysis indicated significant heterogeneity regardless of which study was excluded.

### BMI ≥ 28 kg/m²

**Fig. 3:**
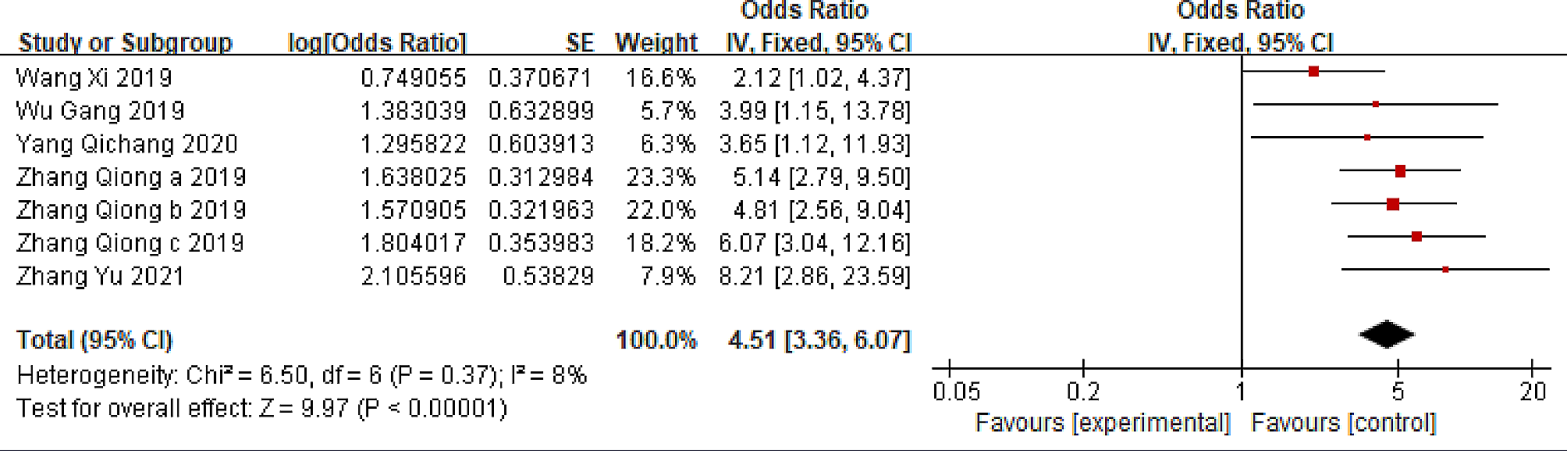
Forest plot of BMI ≥ 28 kg/m²

Five studies evaluating the relationship between BMI ≥ 28 kg/m² and LBP were included [36–39, 41]. The comprehensive findings suggest that individuals in the Chinese population with a BMI of 28 kg/m² or higher are at a heightened risk of experiencing LBP compared with those with a BMI below 28 kg/m² (OR = 4.51; 95% CI: 3.36, 6.07, *P* < 0.00001, I^2^ = 8%) (Fig. 3).

### Female sex

**Fig. 4:**
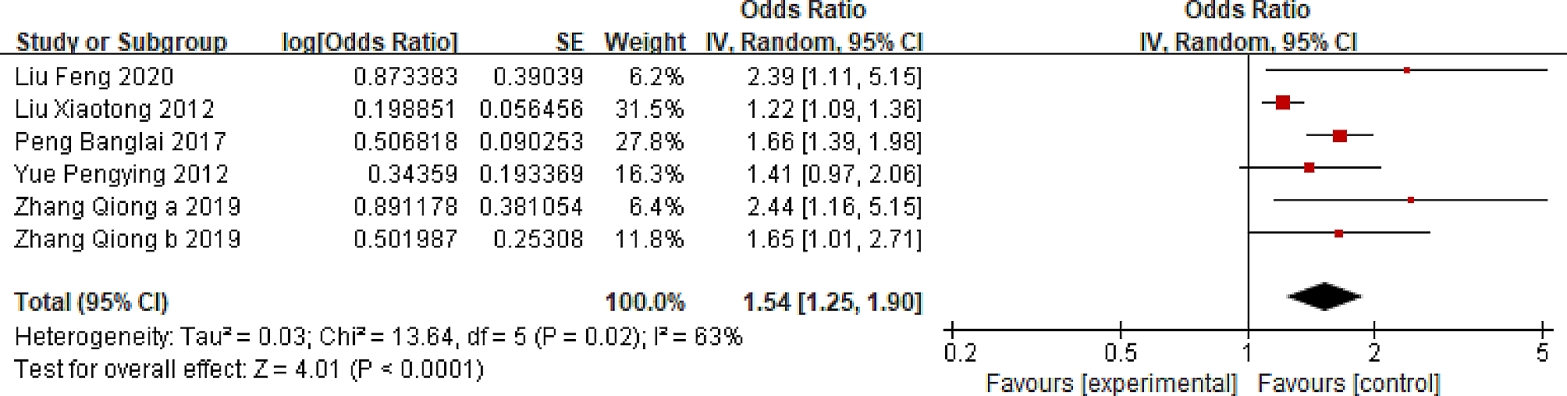
Forest plot of female sex

Five studies assessing the association between women and LBP were included [19, 20, 22, 41, 46]. The comprehensive findings suggest that within the Chinese population, women are more susceptible to LBP than men (OR = 1.54; 95% CI: 1.25, 1.90, *P* < 0.0001, I^2^ = 63%) (Fig. 4). Sensitivity analysis indicated that eliminating Liu et al.’s study could reduce I^2^ to 0% (OR = 1.67; 95% CI: 1.44, 1.93, *P* < 0.00001).

### Vibration exposure at work

**Fig. 5:**
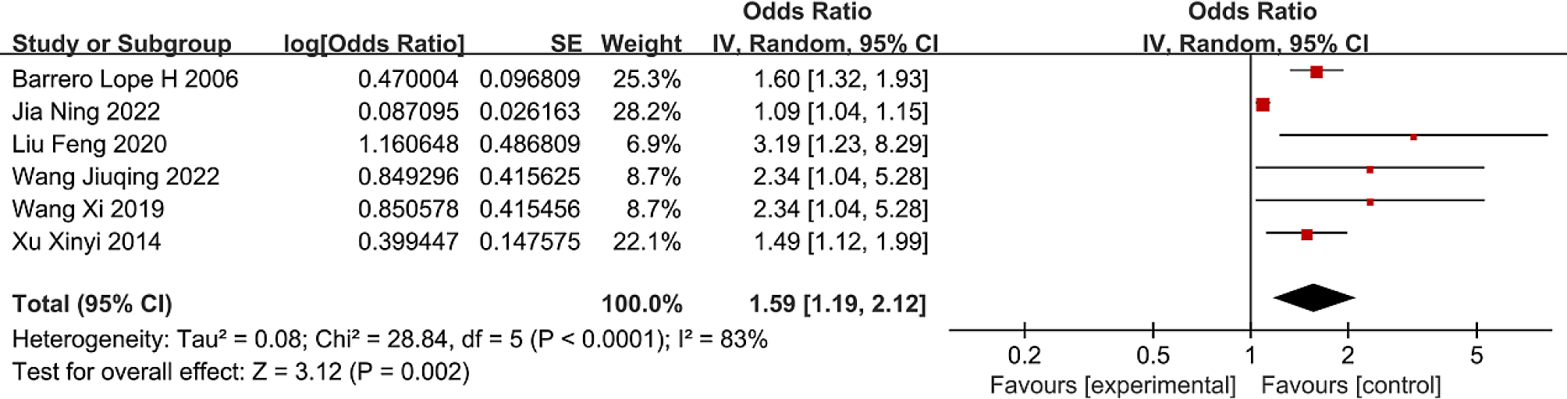
Forest plot of vibration exposure at work

Five studies evaluating the relationship between work-related exposure to vibration sources and LBP were included in the meta-analysis [18, 22, 39, 40, 43]. The findings indicated that individuals in China who were exposed to vibration sources were at a higher risk of experiencing LBP than those not exposed to vibration sources (OR = 1.65; 95% CI: 1.16, 2.34, *P* = 0.006, I^2^ = 84%) (Fig. 5). Sensitivity analysis indicated that eliminating Jia et al.’s study could reduce I^2^ to 8% (OR = 1.70; 95% CI: 1.42, 2.03, *P* < 0.00001). This may be related to Jia’s definition of risk factors, which differs from those of other studies that only mention work involving vibrations. Jia’s study mentioned the use of vibration tools.

### Working overtime

**Fig. 6:**
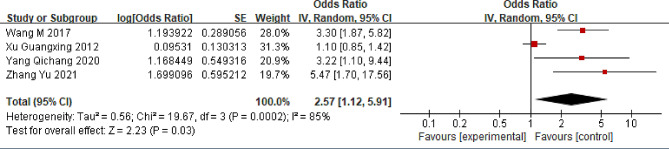
Forest plot of working overtime

Four studies assessing the association between extended working hours and LBP were included in the meta-analysis [36, 38, 42, 44]. These findings suggest that individuals in China who work extended hours are more susceptible to LBP than those who work standard hours (OR = 2.57; 95% CI: 1.12, 5.91, *P* < 0.03, I^2^ = 85%) (Fig. 6). Sensitivity analysis indicated that eliminating the study by Xu et al. could reduce the I^2^ to 0% (OR = 3.55; 95% CI: 2.24, 5.63, *P* < 0.00001).

### Lack of exercise

**Fig. 7:**
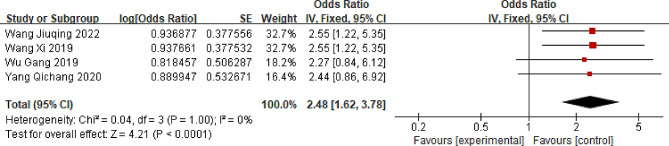
Forest plot of lack of exercise

Four studies assessing the association between exercise and LBP were included in the meta-analysis [37–40]. The comprehensive findings suggest that individuals in China who insufficiently engage in physical activity are at a greater risk of developing LBP than those who exercise regularly (OR = 2.48; 95% CI: 1.62, 3.78, *P* < 0.0001, I^2^ = 0%) (Fig. 7).

### Standing for long periods

**Fig. 8:**
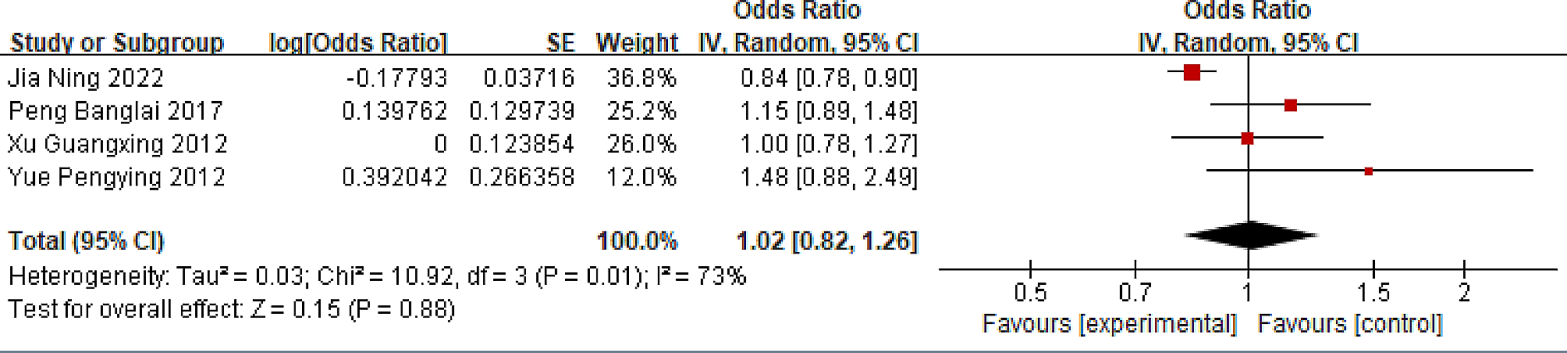
Forest plot of standing for long periods

Four studies assessing the association between prolonged standing and LBP were included [18–20, 44]. The comprehensive findings indicate that prolonged standing is not a risk factor for LBP in the Chinese population (OR = 1.02; 95% CI: 0.82, 1.26, *P* = 0.88, I^2^ = 73%) (Fig. 8). Sensitivity analysis indicated that eliminating Jia’s study could reduce the I^2^ to 0% (OR = 1.10; 95% CI: 0.94, 1.30, *P* = 0.24).

## Discussion

LBP is a common clinical problem in the Chinese population. Limited studies on LBP risk factors exist in China, and their results vary widely. Most studies have only explored the risk factors for LBP in specific occupational and regional populations. For instance, the study participants of Yue et al. [20] and Yao et al. [53] focused on Chinese teachers and adolescents, while Barrero et al. [43] only focused on individuals from rural areas. The risk factors they present have inherent limitations and do not apply to the entire Chinese population. Therefore, we conducted a systematic review to identify the risk factors that may cause LBP in the Chinese population. Seven risk factors were identified: smoking, BMI ≥ 28 kg/m², female sex, vibration exposure at work, working overtime, lack of exercise, and prolonged standing. Among them, prolonged standing was not related to the occurrence of LBP, which may be owing to different occupations and research studies conducted. Wong et al. found that female sex, obesity, and smoking were risk factors for LBP [54]. A LBP study among African school teachers found that lack of exercise and female sex are risk factors for LBP [55]. Jia et al.’s study found that vibration exposure at work and working overtime were risk factors for LBP [18]. These results are consistent with those of the present study.

Smoking and LBP are global public health concerns. Numerous studies [56–58] indicate that smoking increases the risk of LBP. Using a passive smoking model in rats, Nemoto et al. [56] found cracks, tears, and dislocations in the intervertebral discs. A recent study [58] has also shown that smoking and tetramer tryptase accelerate intervertebral disc degeneration by inducing methyltransferase 14-mediated dishevelled-axin domain-containing 1 m6 modifications. Smokers experience significantly greater levels of lumbar pain and dysfunction than non-smokers [57]. The greater the daily tobacco intake, the more pronounced the LBP. Therefore, to prevent the onset of LBP, Chinese individuals are advised to quit smoking or reduce the frequency of smoking.

BMI ≥ 28 kg/m^2^ is a risk factor for LBP in the Chinese population. Adipose tissue due to obesity can release several adipokines (including leptin, resistin, and adiponectin) as well as proinflammatory cytokines such as interleukin-1β, interleukin-6, and tumour necrosis factor-α [59]. Leptin can accelerate the calcification of hyaline cartilage in the cartilaginous endplate, interfering with the transport of nutrients to disc cells [60]. Resistin expression in normal intervertebral discs is low but increases during intervertebral disc degeneration. In intervertebral disc tissues, resistin can activate nuclear factor-κB and p38 mitogen-activated protein kinase, followed by resistin binding to toll-like receptor 4 and increasing chemokine ligand 4 expression. This promotes the infiltration of macrophages [61]. However, the correlation between adiponectin level and LBP remains unclear. All these molecules may cause low-grade inflammation and have been shown to exert detrimental effects on nucleus pulposus and annulus fibrosus cells in vitro [62]. Fortunately, obesity can be modified through exercise and dietary management. Individuals with obesity should consider appropriate weight management strategies. Moreover, there are variations in the risk of LBP across sex groups. Women are at a higher risk of developing LBP than men, and patients with LBP exhibit poor dynamic postural control [63]. This may be attributed to the additional housework that women perform and their relatively low muscle strength. Women, as a group susceptible to LBP, should prioritise enhancing self-management awareness and mitigating LBP stemming from intrinsic factors.

A previous study showed that whole-body vibration can lead to compression, stretching, rotation, and spine flexion, all of which involve back muscles and cause fatigue [64]. Studies of the erector spinae found that electromyography signals increased during whole-body vibration, resulting in a decrease in the signal frequency of muscle fatigue, mainly at the resonance frequency of 5 Hz [65–67]. Kim et al. and Burström et al. concluded that irregular whole-body vibrations are closely associated with musculoskeletal disorders, particularly LBP onset [68, 69]. Moreover, our previous research [70] indicated that vibration therapy remains a viable treatment for LBP, which may be closely related to vibration frequency and amplitude.

The reality many people face is the long working hours they must endure. Individuals are susceptible to musculoskeletal disorders in prolonged and high-intensity work environments, leading to an increased risk of LBP. People who frequently work overtime should be vigilant in preventing and managing this condition. In addition, extended working hours can lead to sedentary behaviour, a fixed posture at work, and prolonged neck forward tilt among professionals working in offices and drivers. A positive correlation exists between sedentary time and two common acute phase reactants, C-reactive protein and fibrinogen [71, 72]. Sedentary behaviour also has a detrimental impact on systemic inflammation [71, 72]. Compared with men, the level of inflammation in women is more influenced by sedentary behaviour [71, 72]. Sedentary behaviour may also reduce bone density, decrease flexibility, and promote weight gain [59]. Furthermore, all participants in this study were adults, and no consideration was given to children. An Iranian meta-analysis [73] suggests that various forms of sedentary behaviour (such as prolonged TV viewing or electronic device use) may also contribute to LBP in children.

This study suggests that a lack of exercise may be a risk factor for LBP. As work and life pressures grow in China, ensuring regular physical activity for industry workers, such as factory employees, office staff, and healthcare professionals, has become challenging. A meta-analysis in China also suggested that Taijiquan, Pilates, sling and core stability exercises can improve LBP symptoms [74]. Taijiquan, a traditional Chinese sport, can enhance joint flexibility and mobility, boost muscle strength and endurance, increase the tensile strength of ligaments and bursae, improve cardiopulmonary function, and alleviate stress and anxiety [75]. In addition, Taijiquan can decrease the expression of serum B-type linalool peptides, enhance blood circulation, increase the metabolism of the lumbar spine, improve the absorption of calcium and other minerals by bone cells, and increase bone density in the lumbar region [76]. We recommend engaging in physical activity as a treatment or preventive measure for LBP.

### Strengths and limitations

To our knowledge, this is the first meta-analysis to explore the risk factors for LBP in the Chinese population. We quantitatively assessed multiple studies on the risk factors for LBP in the Chinese population. With enhanced statistical precision and the resolution of inconsistencies found in previous studies, we arrived at more robust conclusions.

However, this study had inherent limitations. First, the included participants had various occupations and were from different regions of China, leading to heterogeneity in the results. Second, the included studies were cross-sectional, making it impossible to establish a causal relationship between the exposure factors and outcomes. Recall bias was also highly probable. Third, the limited literature included in this study on certain risk factors might have introduced bias into the research outcomes. Lastly, only Chinese and English language literature were included, with no gray literature retrieved, leading to a limited number of articles in the final analysis.

## Conclusion

This study employed evidence-based medical research methods to analyse and explore the risk factors for LBP in the Chinese population. We found that smoking, BMI ≥ 28 kg/m², female sex, vibration exposure at work, working overtime, and lack of exercise may be risk factors for LBP in this population. However, because of the limited number of included studies and their cross-sectional design, causality could not be determined. The certainty of the evidence is also very low. Therefore, these findings should be interpreted with caution. Multicentre, high-quality studies should be conducted in the future. To reduce the prevalence of LBP, the Chinese government and hospitals must develop early screening programs and implement effective preventive and interventional measures.

### Electronic supplementary material

Below is the link to the electronic supplementary material.

Supplementary Material 1

Supplementary Material 2

Supplementary Material 3

Supplementary Material 4

Supplementary Material 5

## Data Availability

The datasets used and analyzed during the current study are available from the corresponding author on reasonable request.

## Abbreviation

LBP: Low back pain
BMI: Body mass index
OR: Odds ratio
95% CI: 95% Confidence interval
