# Supplementary material for: Risk factors for low back pain in the Chinese population: a systematic review and meta-analysis
Source: BMC Public Health. 2024 Apr 26;24:1181. doi: 10.1186/s12889-024-18510-0 (PMC11055313; doi:10.1186/s12889-024-18510-0)
Supplement: Supplementary file 1 — Supplementary Material 1 [file 12889_2024_18510_MOESM1_ESM.docx]

Search strategy in Pubmed

#1 Low Back Pain[MeSH Terms]

#2 Back Pain,Low[Title/Abstract] OR Back Pains,Low[Title/Abstract] OR Low Back Pains[Title/Abstract] OR Pain,Low Back[Title/Abstract] OR Pains,Low Back[Title/Abstract] OR Lumbago[Title/Abstract] OR Lower Back Pain[Title/Abstract] OR Back Pain,Lower[Title/Abstract] OR Back Pains,Lower[Title/Abstract] OR Lower Back Pains[Title/Abstract] OR Pain,Lower Back[Title/Abstract] OR Pains,Lower Back[Title/Abstract] OR Low Back Ache[Title/Abstract] OR Ache,Low Back[Title/Abstract] OR Aches,Low Back[Title/Abstract] OR Back Ache,Low[Title/Abstract] OR Back Aches,Low[Title/Abstract] OR Low Back Aches[Title/Abstract] OR Low Backache[Title/Abstract] OR Backache,Low[Title/Abstract] OR Backaches,Low[Title/Abstract] OR Low Backaches[Title/Abstract] OR Low Back Pain,Postural[Title/Abstract] OR Postural Low Back Pain[Title/Abstract] OR Low Back Pain,Posterior Compartment[Title/Abstract] OR Low Back Pain,Recurrent[Title/Abstract] OR Recurrent Low Back Pain[Title/Abstract] OR Low Back Pain,Mechanical[Title/Abstract] OR Mechanical Low Back Pain[Title/Abstract]

#3 #1OR#2

#4 risk factor*[Title/Abstract] OR influencing factor*[Title/Abstract] OR influence factor*[Title/Abstract] OR contributing factor*[Title/Abstract] OR impact factor*[Title/Abstract] OR relevant factor*[Title/Abstract] OR correlative factor*[Title/Abstract] OR relative factor*[Title/Abstract] OR associated factor*[Title/Abstract] OR predictor*[Title/Abstract]

#5 China[Title/Abstract] OR Chinese[Title/Abstract] OR Hong Kong[Title/Abstract] OR Macao[Title/Abstract] OR Taiwan[Title/Abstract] OR Mainland[Title/Abstract]

#6 #3 AND #4 AND #5

Search strategy in Web of Science

#1 TS=(Low Back Pain OR Back Pain,Low OR Back Pains,Low OR Low Back Pains OR Pain,Low Back OR Pains,Low Back OR Lumbago OR Lower Back Pain OR Back Pain,Lower OR Back Pains,Lower OR Lower Back Pains OR Pain,Lower Back OR Pains,Lower Back OR Low Back Ache OR Ache,Low Back OR Aches,Low Back OR Back Ache,Low OR Back Aches,Low OR Low Back Aches OR Low Backache OR Backache,Low OR Backaches,Low OR Low Backaches OR Low Back Pain,Postural OR Postural Low Back Pain OR Low Back Pain,Posterior Compartment OR Low Back Pain,Recurrent OR Recurrent Low Back Pain OR Low Back Pain,Mechanical OR Mechanical Low Back Pain)

#2 TS=( risk factor* OR influencing factor* OR influence factor* OR contributing factor* OR impact factor* OR relevant factor* OR correlative factor* OR relative factor* OR associated factor* OR predictor*)

#3 TS=(China OR Chinese OR Hong Kong OR Macao OR Taiwan OR Mainland)

#4 #1 AND #2 AND #3

Search strategy in Embase

#1 'low back pain'/exp OR 'low back pain'

#2 ‘Back Pain,Low’:ab,ti OR ‘Back Pains,Low’:ab,ti OR ‘Low Back Pains’:ab,ti OR ‘Pain,Low Back’:ab,ti OR ‘Pains,Low Back’:ab,ti OR ‘Lumbago’:ab,ti OR ‘Lower Back Pain’:ab,ti OR ‘Back Pain,Lower’:ab,ti OR ‘Back Pains,Lower’:ab,ti OR ‘Lower Back Pains’:ab,ti OR ‘Pain,Lower Back’:ab,ti OR ‘Pains,Lower Back’:ab,ti OR ‘Low Back Ache’:ab,ti OR ‘Ache,Low Back’:ab,ti OR ‘Aches,Low Back’:ab,ti OR ‘Back Ache,Low’:ab,ti OR ‘Back Aches,Low’:ab,ti OR ‘Low Back Aches’:ab,ti OR ‘Low Backache’:ab,ti OR ‘Backache,Low’:ab,ti OR ‘Backaches,Low’:ab,ti OR ‘Low Backaches’:ab,ti OR ‘Low Back Pain,Postural’:ab,ti OR ‘Postural Low Back Pain’:ab,ti OR ‘Low Back Pain,Posterior Compartment’:ab,ti OR ‘Low Back Pain,Recurrent’:ab,ti OR ‘Recurrent Low Back Pain’:ab,ti OR ‘Low Back Pain,Mechanical’:ab,ti OR ‘Mechanical Low Back Pain’:ab,ti

#3 #1 OR #2

#4 ‘China’:ab,ti OR ‘Chinese’:ab,ti OR ‘Hong Kong’:ab,ti OR ‘Macao’:ab,ti OR ‘Taiwan’:ab,ti OR ‘Mainland’:ab,ti

#5 ‘risk factor*’:ab,ti OR ‘influencing factor*’:ab,ti OR ‘influence factor*’:ab,ti OR ‘contributing factor*’:ab,ti OR ‘impact factor*’:ab,ti OR ‘relevant factor*’:ab,ti OR ‘correlative factor*’:ab,ti OR ‘relative factor*’:ab,ti OR ‘associated factor*’:ab,ti OR ‘predictor*’:ab,ti

#6 #3 AND #4 AND #5

Search strategy in Cochrane Library

#1 Low Back Pain

#2(Back Pain,Low):ab,ti,kw OR (Back Pains,Low):ab,ti,kw OR (Low Back Pains):ab,ti,kw OR (Pain,Low Back):ab,ti,kw OR (Pains,Low Back):ab,ti,kw OR (Lumbago):ab,ti,kw OR (Lower Back Pain):ab,ti,kw OR (Back Pain,Lower):ab,ti,kw OR (Back Pains,Lower):ab,ti,kw OR (Lower Back Pains):ab,ti,kw OR (Pain,Lower Back):ab,ti,kw OR (Pains,Lower Back):ab,ti,kw OR (Low Back Ache):ab,ti,kw OR (Ache,Low Back):ab,ti,kw OR (Aches,Low Back):ab,ti,kw OR (Back Ache,Low):ab,ti,kw OR (Back Aches,Low):ab,ti,kw OR (Low Back Aches):ab,ti,kw OR (Low Backache):ab,ti,kw OR (Backache,Low):ab,ti,kw OR (Backaches,Low):ab,ti,kw OR (Low Backaches):ab,ti,kw OR (Low Back Pain,Postural):ab,ti,kw OR (Postural Low Back Pain):ab,ti,kw OR (Low Back Pain,Posterior Compartment):ab,ti,kw OR (Low Back Pain,Recurrent):ab,ti,kw OR (Recurrent Low Back Pain):ab,ti,kw OR (Low Back Pain,Mechanical):ab,ti,kw OR (Mechanical Low Back Pain):ab,ti,kw

#3 #1 OR #2

#4 (China):ab,ti,kw OR (Chinese):ab,ti,kw OR (Hong Kong):ab,ti,kw OR (Macao):ab,ti,kw OR (Taiwan):ab,ti,kw OR (Mainland):ab,ti,kw

#5 (risk factor*):ab,ti,kw OR (influencing factor*):ab,ti,kw OR (influence factor*):ab,ti,kw OR (contributing factor*):ab,ti,kw OR (impact factor*):ab,ti,kw OR (relevant factor*):ab,ti,kw OR (correlative factor*):ab,ti,kw OR (relative factor*):ab,ti,kw OR (associated factor*):ab,ti,kw OR (predictor*):ab,ti,kw

#6 #3 AND #4 AND #5
