# Supplementary material for: Risk factors for low back pain in the Chinese population: a systematic review and meta-analysis
Source: BMC Public Health. 2024 Apr 26;24:1181. doi: 10.1186/s12889-024-18510-0 (PMC11055313; doi:10.1186/s12889-024-18510-0)
Supplement: Supplementary file 2 — Supplementary Material 2 [file 12889_2024_18510_MOESM2_ESM.docx]

Studies excluded in the last step of the systematic search of the literature (n=82)

1. Zhu HP, Qi H, Liu XH, et al. The prevalence of disability and associated factors among community adults in the baseline of CHCN-BTH Cohort Study. BMC Public Health. 2023;23(1):1727. Published 2023 Sep 5. doi:10.1186/s12889-023-15066-3
2. Wang Z, Zhao X, Li Y, et al. Development and validation of a multimodal feature fusion prognostic model for lumbar degenerative disease based on machine learning: a study protocol. BMJ Open. 2023;13(9):e072139. Published 2023 Sep 5. doi:10.1136/bmjopen-2023-072139
3. Shi Z, Yan F, Lu Y, Liu W, Wang Z, Zhang H. Pregnancy-related low back/pelvic girdle pain: Prevalence, severity, and risk factors in Zhengzhou, China. J Back Musculoskelet Rehabil. 2023;36(4):895-902. doi:10.3233/BMR-220147
4. Liu Q, Liu X, Lin H, et al. Occupational low back pain prevention capacity of nurses in China: A multicenter cross-sectional study. Front Public Health. 2023;11:1103325. Published 2023 Mar 16. doi:10.3389/fpubh.2023.1103325
5. Zhang G, Gao L, Zhang D, et al. Mawangdui-Guidance Qigong Exercise for patients with chronic non-specific low back pain: Study protocol of a randomized controlled trial. Front Neurosci. 2023;17:1090138. Published 2023 Mar 13. doi:10.3389/fnins.2023.1090138
6. Li Z, Gao X, Ding W, Li R, Yang S. Asymmetric distribution of Modic changes in patients with lumbar disc herniation. Eur Spine J. 2023;32(5):1741-1750. doi:10.1007/s00586-023-07664-x
7. Huang Z, Chen J, Su Y, et al. Impact of dyslipidemia on the severity of symptomatic lumbar spine degeneration: A retrospective clinical study. Front Nutr. 2022;9:1033375. Published 2022 Dec 13. doi:10.3389/fnut.2022.1033375
8. Lin Y, Zhang X, Li H, Huang Y, Zhang W, Zhang C. Musculoskeletal pain is prevalent in Chinese medical and dental students: A cross-sectional study. Front Public Health. 2022;10:1046466. Published 2022 Nov 24. doi:10.3389/fpubh.2022.1046466
9. He C, Chen H, Guo L, et al. Prevalence and factors associated with comorbid depressive symptoms among people with low back pain in China: A cross-sectional study. Front Psychiatry. 2022;13:922733. Published 2022 Jul 25. doi:10.3389/fpsyt.2022.922733
10. Wang M, Ding Q, Sang L, Song L. Prevalence of Pain and Its Risk Factors Among ICU Personnel in Tertiary Hospital in China: A Cross-Sectional Study. J Pain Res. 2022;15:1749-1758. Published 2022 Jun 20. doi:10.2147/JPR.S366536
11. Hu Y, Yang Z, Li Y, et al. Prevalence and Associated Factors of Depressive Symptoms Among Patients With Chronic Low Back Pain: A Cross-Sectional Study. Front Psychiatry. 2022;12:820782. Published 2022 Jan 13. doi:10.3389/fpsyt.2021.820782
12. Ma K, Zhuang ZG, Wang L, et al. The Chinese Association for the Study of Pain (CASP): Consensus on the Assessment and Management of Chronic Nonspecific Low Back Pain. Pain Res Manag. 2019;2019:8957847. Published 2019 Aug 15. doi:10.1155/2019/8957847
13. Zhou M, Wang H, Zeng X, et al. Mortality, morbidity, and risk factors in China and its provinces, 1990-2017: a systematic analysis for the Global Burden of Disease Study 2017 [published correction appears in Lancet. 2020 Jul 4;396(10243):26]. Lancet. 2019;394(10204):1145-1158. doi:10.1016/S0140-6736(19)30427-1
14. Hu XJ, Chen LH, Battié MC, Wang Y. Methodology and cohort profile for the Hangzhou Lumbar Spine Study: a study focusing on back health in a Chinese population. J Zhejiang Univ Sci B. 2018;19(7):547-558. doi:10.1631/jzus.B1700484
15. Du S, Hu L, Bai Y, et al. The Influence of Self-Efficacy, Fear-Avoidance Belief, and Coping Styles on Quality of Life for Chinese Patients with Chronic Nonspecific Low Back Pain: A Multisite Cross-Sectional Study. Pain Pract. 2018;18(6):736-747. doi:10.1111/papr.12660
16. Wáng YXJ, Deng M, Griffith JF, et al. Lumbar Spondylolisthesis Progression and De Novo Spondylolisthesis in Elderly Chinese Men and Women: A Year-4 Follow-up Study. Spine (Phila Pa 1976). 2016;41(13):1096-1103. doi:10.1097/BRS.0000000000001507
17. Stewart Williams J, Ng N, Peltzer K, et al. Risk Factors and Disability Associated with Low Back Pain in Older Adults in Low- and Middle-Income Countries. Results from the WHO Study on Global AGEing and Adult Health (SAGE). PLoS One. 2015;10(6):e0127880. Published 2015 Jun 4. doi:10.1371/journal.pone.0127880
18. Wang S, Kou C, Liu Y, et al. Rural-urban differences in the prevalence of chronic disease in northeast China. Asia Pac J Public Health. 2015;27(4):394-406. doi:10.1177/1010539514551200
19. Mu J, Ge W, Zuo X, Chen Y, Huang C. Analysis of association between IL-1β, CASP-9, and GDF5 variants and low-back pain in Chinese male soldier: clinical article. J Neurosurg Spine. 2013;19(2):243-247. doi:10.3171/2013.4.SPINE12782
20. Law RK, Lee EW, Law SW, Chan BK, Chen PP, Szeto GP. The predictive validity of OMPQ on the rehabilitation outcomes for patients with acute and subacute non-specific LBP in a Chinese population. J Occup Rehabil. 2013;23(3):361-370. doi:10.1007/s10926-012-9404-y
21. Yeung SS. Factors contributing to work related low back pain among personal care workers in old age. Work. 2012;41 Suppl 1:1873-1883. doi:10.3233/WOR-2012-0401-1873
22. Yeung SS, Yuan J. Low back pain among personal care workers in an old age home: work-related and individual factors. AAOHN J. 2011;59(8):345-353. doi:10.3928/08910162-20110726-01
23. Mok LC, Lee IF. Anxiety, depression and pain intensity in patients with low back pain who are admitted to acute care hospitals. J Clin Nurs. 2008;17(11):1471-1480. doi:10.1111/j.1365-2702.2007.02037.x
24. Horng YS, Hwang YH, Wu HC, et al. Predicting health-related quality of life in patients with low back pain. Spine (Phila Pa 1976). 2005;30(5):551-555. doi:10.1097/01.brs.0000154623.20778.f0
25. Ge ZM, Zhen XH, Wang DG, et al. Incidence and influencing factors of low back pain in officers and soldiers of long-distance ships. Journal of Navy Medicine. 2022,43(10):1069-1073.
26. Yuan L, Huang Z, Han W, et al. The impact of dyslipidemia on lumbar intervertebral disc degeneration and vertebral endplate modic changes: a cross-sectional study of 1035 citizens in China. BMC Public Health. 2023;23(1):1302. doi:10.1186/s12889-023-16224-3
27. Chunmei D, Yong C, Long G, Mingsheng T, Hua L, Ping Y. Self efficacy associated with regression from pregnancy-related pelvic girdle pain and low back pain following pregnancy. BMC Pregnancy Childbirth. 2023;23(1):122. Published 2023 Feb 21. doi:10.1186/s12884-023-05393-z
28. Wu D, Wong P, Guo C, Tam LS, Gu J. Pattern and trend of five major musculoskeletal disorders in China from 1990 to 2017: findings from the Global Burden of Disease Study 2017. BMC Med. 2021;19(1):34. Published 2021 Feb 4. doi:10.1186/s12916-021-01905-w
29. Feng Q, Liu S, Yang L, Xie M, Zhang Q. The Prevalence of and Risk Factors Associated with Musculoskeletal Disorders among Sonographers in Central China: A Cross-Sectional Study. PLoS One. 2016;11(10):e0163903. Published 2016 Oct 3. doi:10.1371/journal.pone.0163903
30. Mok FP, Samartzis D, Karppinen J, Fong DY, Luk KD, Cheung KM. Modic changes of the lumbar spine: prevalence, risk factors, and association with disc degeneration and low back pain in a large-scale population-based cohort. Spine J. 2016;16(1):32-41. doi:10.1016/j.spinee.2015.09.060
31. Chang HY, Lai YH, Jensen MP, et al. Factors associated with low back pain changes during the third trimester of pregnancy. J Adv Nurs. 2014;70(5):1054-1064. doi:10.1111/jan.12258
32. Cho CY, Hwang YS, Cherng RJ. Musculoskeletal symptoms and associated risk factors among office workers with high workload computer use. J Manipulative Physiol Ther. 2012;35(7):534-540. doi:10.1016/j.jmpt.2012.07.004
33. Kwok AW, Gong JS, Wang YX, et al. Prevalence and risk factors of radiographic vertebral fractures in elderly Chinese men and women: results of Mr. OS (Hong Kong) and Ms. OS (Hong Kong) studies. Osteoporos Int. 2013;24(3):877-885. doi:10.1007/s00198-012-2040-8
34. Sun ZM, Ling M, Chang YH, et al. Nan Fang Yi Ke Da Xue Xue Bao. 2010;30(11):2488-2491.
35. Zhang YG, Sun Z, Zhang Z, Liu J, Guo X. Risk factors for lumbar intervertebral disc herniation in Chinese population: a case-control study. Spine (Phila Pa 1976). 2009;34(25):E918-E922. doi:10.1097/BRS.0b013e3181a3c2de
36. Szeto GP, Lam P. Work-related musculoskeletal disorders in urban bus drivers of Hong Kong. J Occup Rehabil. 2007;17(2):181-198. doi:10.1007/s10926-007-9070-7
37. Yip YB, Ho SC, Chan SG. Tall stature, overweight and the prevalence of low back pain in Chinese middle-aged women. Int J Obes Relat Metab Disord. 2001;25(6):887-892. doi:10.1038/sj.ijo.0801557
38. Weng FB, Zhou JX, Yang LW, et al. Incidence and risk factors of persistent low back pain after posterior decompression and fusion. Chinese Journal of Orthopaedic Surgery. 2019,27(21):1921-1926.
39. Yao FH, Zhan XL, Liu C, et al. Analysis of risk factors of chronic low back pain in women with lumbar degenerative disease after surgery. Chinese Spinal Cord Journal, 2017,27(12):1071-1080.
40. Li SN, He XB. Analysis of risk factors for lower back pain complicating postoperative lumbar disc herniation. Journal of North Sichuan Medical College,2013,28(03):280-282.
41. Mao SL. Influencing factors of chronic low back pain in patients after thoracolumbar compression fracture surgery. Chinese Journal of Minkang Medicine,2022,34(07):4-7.
42. Ma T, Shen MF, Li CX, et al. Risk factors of low back pain after lumbar puncture in adult patients in Department of Neurosurgery. General Nursing, 2021,19(18):2567-2570.
43. Fan XX. Influencing factors of low back pain in patients with lumbar spinal stenosis after transforaminal endoscopic treatment. Liaoning Medical Journal,2021,35(01):32-35.
44. Weng WQ, Xu HX, Zhang ZP, et al. Related factors and biomechanical characteristics of lumbar facet joint degeneration. Chinese Journal of Tissue Engineering Research,2020,24(24):3883-3889.
45. Ye LP, Yang XJ, Wang XJ, et al. Analysis of factors associated with low back pain after lower limb vascular intervention. General Nursing,2019,17(27):3349-3351.
46. Ren XM. Prevalence of low back pain and related influencing factors among residents in Xiushan County, Chongqing. Shandong University,2023. DOI:10.27272/d.cnki.gshdu.2023.006437
47. Yao WG. Risk factors of nonspecific low back pain in school children and adolescents. Southern Medical University, 2011.
48. Liu K. Epidemiological investigation of neck and low back pain and shoulder and knee joint injuries in land aviation helicopter pilots. Chinese People's Liberation Army Medical College,2014.
49. Wang XW. Epidemiological investigation of osteogenic low back pain in armored infantry and experimental study of related serum inflammatory factors. Xinxiang Medical College, 2013.
50. Luo JZ. Risk factors analysis, early warning model construction and treatment of spinal surgical site infection. Southern Medical University, 2022.DOI:10.27003/d.cnki.gojyu.2022.001042
51. Zhao CY. Multivariate correlation analysis based on MRI paravertebral muscle morphology and risk factors of low back pain. Kunming Medical University,2019.DOI:10.27202/d.cnki.gkmyc.2019.000384
52. Tang GM, Wang LY, Chen B, et al. Risk factors of acute low back pain after percutaneous kyphoplasty for osteoporotic vertebral compression fractures[C]// Bone injury rehabilitation professional committee of China Rehabilitation Medicine Association. Abstracts of the first academic annual meeting of the bone and trauma rehabilitation professional committee of the Chinese Association of Rehabilitation Medicine. 904 Hospital of the Joint Service Support Force of the Chinese People's Liberation Army,2023:2.DOI:10.26914/c.cnkihy.2023.009829
53. Wang HY, Feng YT, Wang JJ, Lim SW, Ho CH. Incidence of low back pain and potential risk factors among pharmacists: A population-based cohort study in Taiwan. Medicine (Baltimore). 2021;100(9):e24830. doi:10.1097/MD.0000000000024830
54. Mei Q, Li C, Yin Y, Wang Q, Wang Q, Deng G. The relationship between the psychological stress of adolescents in school and the prevalence of chronic low back pain: a cross-sectional study in China. Child Adolesc Psychiatry Ment Health. 2019;13:24. Published 2019 Jun 17. doi:10.1186/s13034-019-0283-2
55. Feng Q, Jiang C, Zhou Y, Huang Y, Zhang M. Relationship between spinal morphology and function and adolescent non-specific back pain: A cross-sectional study. J Back Musculoskelet Rehabil. 2017;30(3):625-633. doi:10.3233/BMR-160544
56. Hou ZH, Shi JG, Ye H, et al. Prevalence of low back pain among soldiers at an army base. Chin Med J (Engl). 2013;126(4):679-682.
57. Yao W, Luo C, Ai F, Chen Q. Risk factors for nonspecific low-back pain in Chinese adolescents: a case-control study. Pain Med. 2012;13(5):658-664. doi:10.1111/j.1526-4637.2012.01369.x
58. Lin PH, Tsai YA, Chen WC, Huang SF. Prevalence, characteristics, and work-related risk factors of low back pain among hospital nurses in Taiwan: a cross-sectional survey. Int J Occup Med Environ Health. 2012;25(1):41-50. doi:10.2478/s13382-012-0008-8
59. Cheung K. The incidence of low back problems among nursing students in Hong Kong. J Clin Nurs. 2010;19(15-16):2355-2362. doi:10.1111/j.1365-2702.2009.03091.x
60. Feng CK, Chen ML, Mao IF. Prevalence of and risk factors for different measures of low back pain among female nursing aides in Taiwanese nursing homes. BMC Musculoskelet Disord. 2007;8:52. Published 2007 Jun 25. doi:10.1186/1471-2474-8-52
61. Yip YB, Ho SC, Chan SG. Identifying risk factors for low back pain (LBP) in Chinese middle-aged women: a case-control study. Health Care Women Int. 2004;25(4):358-369. doi:10.1080/07399330490278367
62. Yip YB, Ho SC, Chan SG. Socio-psychological stressors as risk factors for low back pain in Chinese middle-aged women. J Adv Nurs. 2001;36(3):409-416. doi:10.1046/j.1365-2648.2001.01988.x
63. Wang H, Yang HJ. Analysis of the causes of non-specific changes in College Students -- a survey of risk factors for non-specific low back pain. Journal of Xi An Jiaotong university (Medical Edition), 2023,44(02):185-188.
64. Ma YG, Zhang DW, Shangguan L, et al. Investigation and risk factors analysis of neck and low back pain in pilots of different aircraft types. Journal of Air Force Medicine, 2021,37 (06): 465-468+480
65. Fan B, Luo L. Investigation and risk factors analysis of non-specific low back pain in high school students. Journal of neck and low back pain, 2019,40 (05): 712-713.
66. Fei A, Xu JH, Song XL, et al. Analysis of inducing factors of low back pain in submarine officers and soldiers. Journal of Naval Medicine, 2018,39 (03): 193-195+201
67. Zhang S, Chen HR, An SS, et al. Risk factors of low back pain in fighter pilots. Journal of the Third Military Medical University, 2015,37 (24): 2481-2485.doi:10.16016/j.1000-5404.201504065
68. Yao WG, Zheng YT, Cui QQ, et al. Epidemiological investigation on risk factors of nonspecific low back pain in school students. Public health and preventive medicine, 2014,25 (04): 14-16+20
69. Cao RQ, Yang JC, Yu M. Epidemiological survey and influencing factors of low back pain among high school students in Foshan. Chinese School Medicine, 2020,34 (08): 576-579+591.
70. Su W, Guo YH, Niu ZY, et al. Risk factors of nonspecific low back pain in soldiers of armed police force in Shanxi Province. Chinese Journal of Medicine and Clinic, 2020,20 (18): 3010-3011.
71. Lu H, Chen F, Yan Q, et al. Correlation between low back pain of liver and kidney deficiency type and sex hormones in elderly Zhuang men. Liaoning Journal of Traditional Chinese Medicine, 2020,47 (04): 8-11.doi:10.13192/j.issn.1000-1719.2020.04.003.
72. Zhang MX, Shen LL, He Li, et al. Investigation on the cognitive status of patients with chronic neck and low back pain on occupational musculoskeletal diseases. Industrial health and occupational diseases, 2019,45 (04): 275-278.doi:10.13692/j.cnki.gywsyzyb.2019.04.010
73. Fang YW, Wu YL,Liao NN, et al. Epidemiological investigation and prevention suggestions of neck and low back pain in a fighter pilot in Xinjiang. Southwest national defense medicine, 2018,28 (03): 299-301
74. Heidarimoghadam R, Mohammadi Y, Kordi R. Effects of Biopsychosocial Interventions on Non-specific Chronic Low Back Pain and Its Related Disabilities among Students. J Res Health Sci. 2022, 22(4):e00568. doi: 10.34172/jrhs.2022.103.
75. Morham S, Reichardt A, Toth A, Olin G, Pohlman K, Passmore SR. Patient Characteristics and Clinical Outcomes Associated With Conservative Treatment for Spine Pain in Women Experiencing Socioeconomic Challenges. J Manipulative Physiol Ther. 2022, 45(9):633-640. doi: 10.1016/j.jmpt.2023.04.001.
76. Zhu YL, He XQ, Chen HD, et al. Epidemiological study on low back pain in soldiers. Northwest Journal of Defense Medicine, 2010,31 (01): 7-9.doi:10.16021/j.cnki.1007-8622.2010.01.052
77. Yang K, Xu J, Liu JZ. Logistic regression analysis of risk factors for low back pain in office workers. Chin Occup Med, 2000; 27(5): 58.
78. Yang ZF. Behavioral factors and risk factors of adults with low back pain. J Cervic Lumb, 2020; 41(2): 199-204.
79. Li JY , Wang S, He LH, Wu SS, Yang L, Y u SF, et al. Risk factors of low back pain among the Chinese occupational population: a case-control study. Biomed Environ Sci. 2012; 25(4):421-429.
80. Xu XY , Qiu SJ, An SL, Jin AM, Min SX. Analysis of risk factors of nonspecific low back pain in a community population: a case-control study. J South Med Univ, 2014; 34(12): 1794-1798.
81. Ren XM, Bai DQ, Zhang YA, Lin HD, Zhang S, Li DY, et al. Residents of mountainous areas have a higher low back pain prevalence than flat areas of Chongqing, china: a cross-sectional study. J Pain Res,2023(16): 1169–1183.
82. Ye SY, Jing QL,Chen W, Lu J. Risk factors of non-specific neck pain and low back pain in computer-using office workers in China: a cross-sectional study. BMJ Open, 2017(7):e014914.
