# Supplementary material for: Risk factors for low back pain in the Chinese population: a systematic review and meta-analysis
Source: BMC Public Health. 2024 Apr 26;24:1181. doi: 10.1186/s12889-024-18510-0 (PMC11055313; doi:10.1186/s12889-024-18510-0)
Supplement: Supplementary file 3 — Supplementary Material 3 [file 12889_2024_18510_MOESM3_ESM.docx]

Risk of bias evaluation results of included cross-sectional studies

| References | Selection Bias | Performance bias | Measuring bias | Attrition bias | Reporting bias | Quality score |
| --- | --- | --- | --- | --- | --- | --- |
| Zhang Yu 2021 | 2 | 1 | 1 | 1 | 3 | 8 |
| Wu Gang 2019 | 2 | 1 | 1 | 1 | 3 | 8 |
| Yang Qichang 2020 | 3 | 1 | 1 | 1 | 2 | 8 |
| Wang Xi 2019 | 3 | 1 | 1 | 1 | 3 | 9 |
| Liu Feng 2020 | 3 | 1 | 1 | 1 | 3 | 9 |
| Wang Jiuqing 2022 | 3 | 1 | 1 | 1 | 2 | 8 |
| Peng Banglai 2017 | 3 | 1 | 1 | 0 | 3 | 8 |
| Jia Ning 2022 | 3 | 1 | 1 | 1 | 3 | 9 |
| Zhang Qiong a 2019 | 3 | 1 | 1 | 1 | 3 | 9 |
| Zhang Qiong b 2019 | 3 | 1 | 1 | 1 | 3 | 9 |
| Zhang Qiong c 2019 | 3 | 1 | 1 | 1 | 3 | 9 |
| Wang M 2017 | 4 | 1 | 1 | 1 | 2 | 9 |
| Yue Pengying 2012 | 3 | 1 | 1 | 1 | 3 | 9 |
| Barrero Lope H 2006 | 4 | 1 | 1 | 1 | 3 | 10 |
| Xu Guangxing 2012 | 3 | 1 | 1 | 1 | 3 | 9 |
| Wei Gejin 2018 | 2 | 1 | 1 | 1 | 3 | 8 |
| Liu Xiaotong 2012 | 3 | 1 | 1 | 1 | 2 | 8 |
