# Supplementary material for: Risk factors for low back pain in the Chinese population: a systematic review and meta-analysis
Source: BMC Public Health. 2024 Apr 26;24:1181. doi: 10.1186/s12889-024-18510-0 (PMC11055313; doi:10.1186/s12889-024-18510-0)
Supplement: Supplementary file 4 — Supplementary Material 4 [file 12889_2024_18510_MOESM4_ESM.docx]

**Certainty of Evidence Assessment**

| Risk factors | Study Design | Risk of bias | Inconsistency | Indirectness | Imprecision | Other considerations | Certainty |
| --- | --- | --- | --- | --- | --- | --- | --- |
|  |  |  |  |  |  |  |  |
| Cigarette smoking | Observational  studies | Serious | Serious | Not serious | Not serious | None | Very Low |
| BMI ≥ 28kg/m² | Observational  studies | Serious | Not Serious | Not serious | Not serious | None | Very Low |
| Female sex | Observational  studies | Serious | Serious | Not serious | Not serious | None | Very Low |
| Vibration exposure at work | Observational  studies | Serious | Serious | Not serious | Not serious | None | Very Low |
| Working overtime | Observational  studies | Serious | Serious | Not serious | Not serious | None | Very Low |
| Lack of exercise | Observational  studies | Serious | Not Serious | Not serious | Not serious | None | Very Low |
| Standing for long periods | Observational  studies | Serious | Serious | Not serious | Not serious | None | Very Low |
