# Supplementary material for: Risk factors for low back pain in the Chinese population: a systematic review and meta-analysis
Source: BMC Public Health. 2024 Apr 26;24:1181. doi: 10.1186/s12889-024-18510-0 (PMC11055313; doi:10.1186/s12889-024-18510-0)

**Begg's test and Egger's test for the risk factors of LBP in the Chinese population**

| Risk Factors | Begg’s Test | | Egger’s Test | | |
| --- | --- | --- | --- | --- | --- |
|  | Z | P | t | P | 95%CI |
| Cigarette smoking | 2.07 | 0.06 | 5.63 | 0.005 | 0.97~2.86 |
| BMI ≥ 28kg/m² | -0.45 | 0.764 | -0.02 | 0.984 | -4.33~4.26 |
| Female sex | 0.94 | 0.452 | 2.15 | 0.098 | -0.53~4.15 |
| Vibration exposure at work | 0.49 | 0.806 | 3.58 | 0.037 | 0.29~4.90 |
| Working overtime | 0.68 | 0.734 | 2.95 | 0.099 | -1.67~8.91 |
| Lack of exercise | -1.36 | 0.308 | -2.09 | 0.172 | -1.70~0.59 |
| Standing for long periods | 1.36 | 0.308 | 6.42 | 0.023 | 0.87~4.40 |

Cigarette smoking


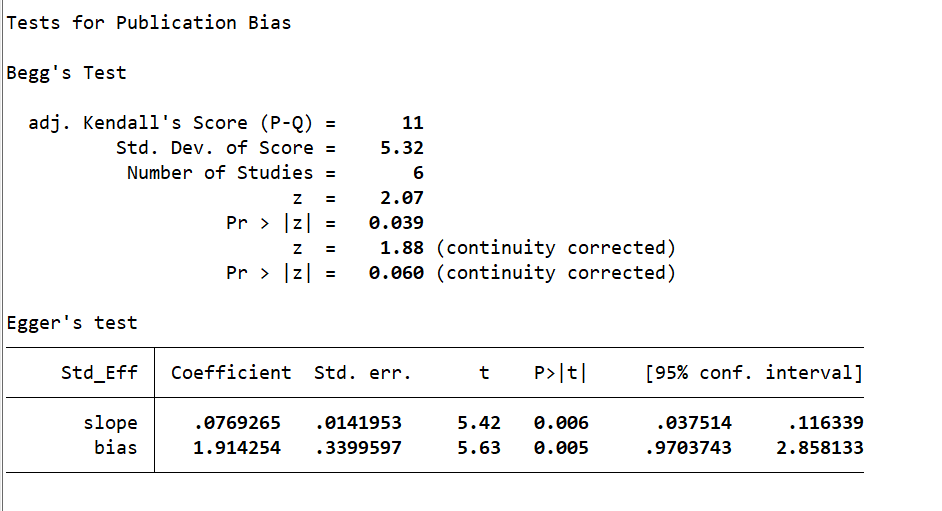


BMI ≥ 28kg/m²


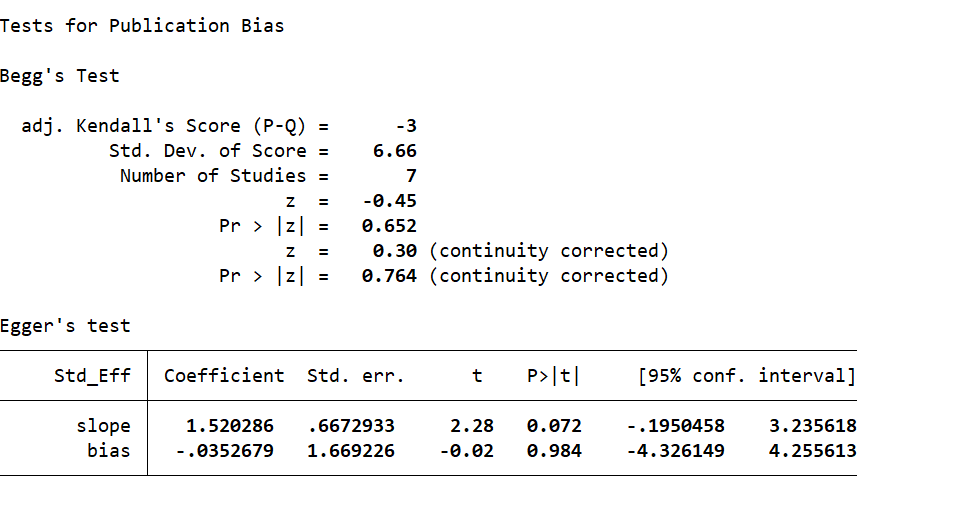


Female sex


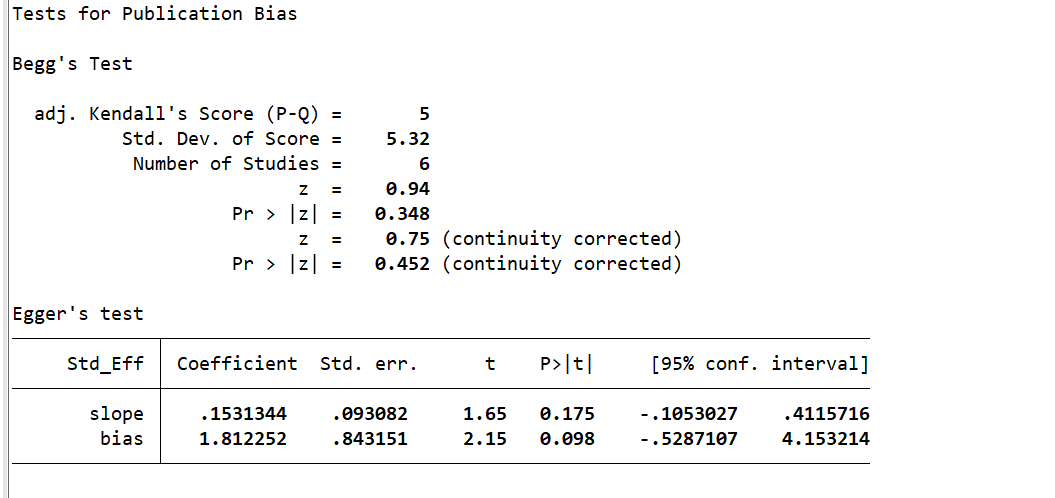


Vibration exposure at work


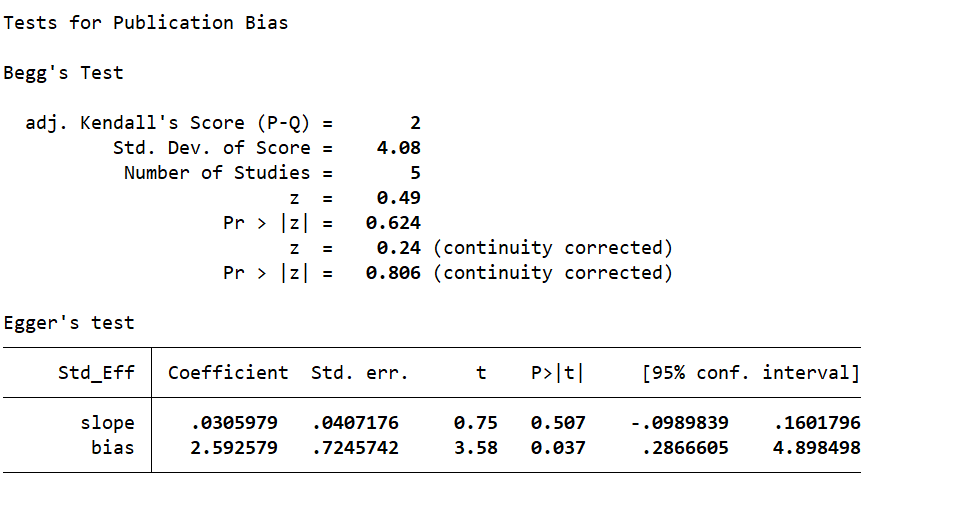


Working overtime


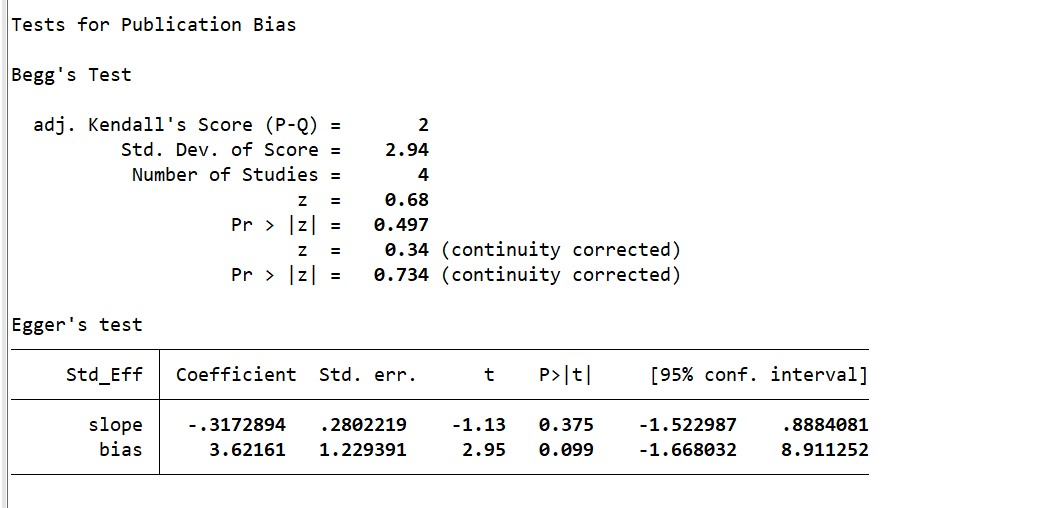


Lack of exercise


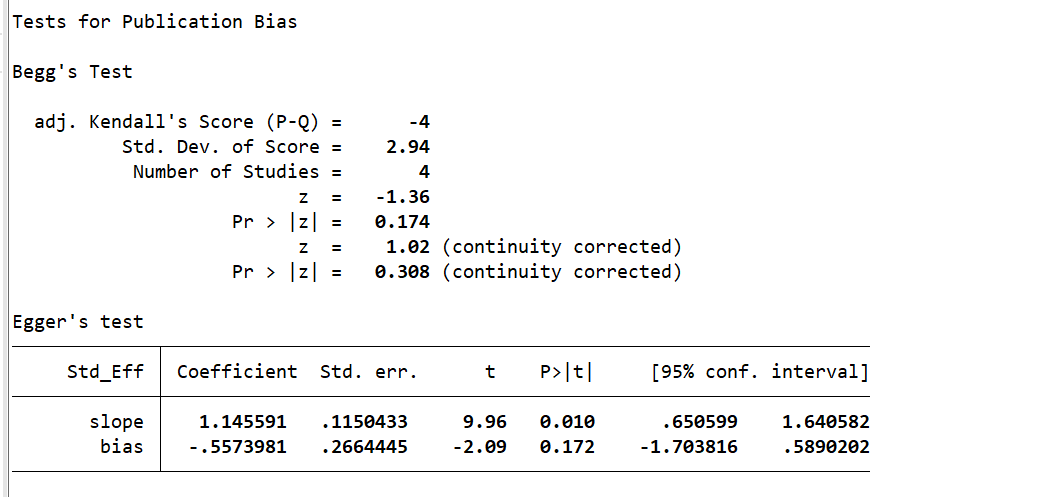


Standing for long periods


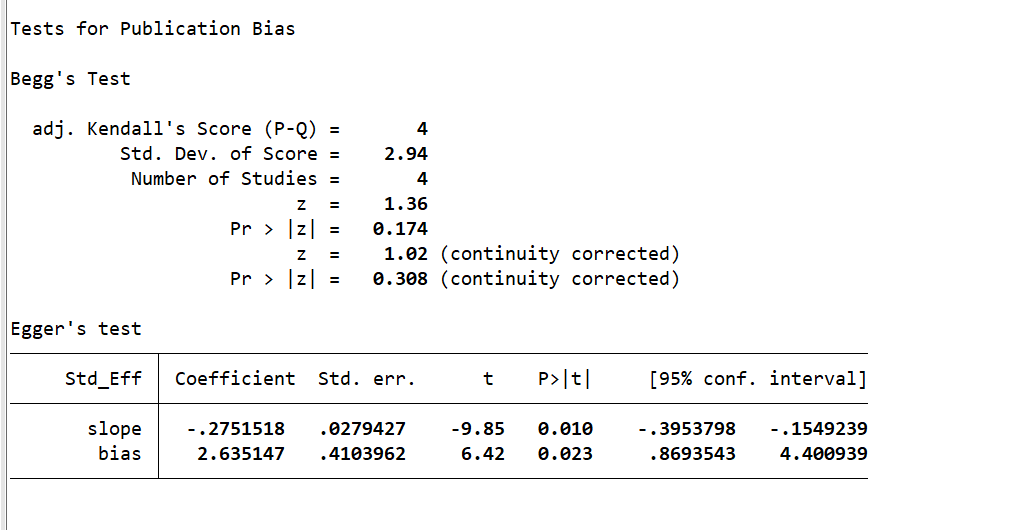

Supplement: Supplementary file 5 — Supplementary Material 5 [file 12889_2024_18510_MOESM5_ESM.doc]
